# A Non-Invasive Technique to Unveil Renal Implications in Anderson–Fabry Disease

**DOI:** 10.3390/biomedicines12091950

**Published:** 2024-08-26

**Authors:** Matteo Gravina, Dario Troise, Barbara Infante, Luciano Tartaglia, Bruno Minopoli, Costanza Allegra, Grazia Casavecchia, Marcella Gambacorta, Carmen Montanile, Silvia Mercuri, Luca Macarini, Giovanni Stallone

**Affiliations:** 1 Radiology Unit, Department of Medical and Surgical Sciences, University of Foggia, 71122 Foggia, Italy; 2 Nephrology, Dialysis and Transplantation Unit, Advanced Research Center on Kidney Aging (A.R.K.A.), Department of Medical and Surgical Sciences, University of Foggia, 71122 Foggia, Italy; 3 Renal Medicine and Baxter Novum, Department of Clinical Science, Intervention and Technology, Karolinska Institutet, 14152 Stockholm, Sweden; 4 Cardiology Unit, Department of Medical and Surgical Sciences, University Hospital of Foggia, University of Foggia, 71122 Foggia, Italy

**Keywords:** Anderson–Fabry disease, magnetic resonance, chronic kidney disease, glycosphingolipid accumulation, genetic disease

## Abstract

Background: Anderson–Fabry disease (AFD) is a rare genetic disorder characterized by a deficiency of α-galactosidase A activity and the accumulation of glycosphingolipids in tissues, which leads to multiorgan damage. Cardiovascular magnetic resonance (CMR) and the T1 mapping technique are essential tools for the assessment of AFD cardiac involvement. Moreover, the T1 mapping technique has proved to be a successful non-invasive method for the early detection of patients most at risk for kidney disease. We evaluated the application of MRI in patients with AFD to assess renal involvement. Methods: We conducted a retrospective analysis of 19 patients (Group A) with histologically proven AFD who underwent routine CMR examinations for the evaluation of cardiac involvement, selecting specific sequences that also showed the left kidney, compared to a control population (Group B, 19 patients) without kidney disease. A Spearman’s rank-order correlation was run to assess the relationship between the T1 mapping values of the heart and kidney in Group A and between the kidneys of Groups A and B. Results: There was a positive correlation between the heart and kidney T1 values in Group A (rho = 0.32). More interestingly, we observed a negative correlation between the kidney values of both groups (Group A mean 1284 ± 137 ms, Group B mean 1073 ± 57 ms, rho = −0.38), which is probably related to the presence of microvascular damage and infiltrates in the kidneys of AFD patients. Conclusions: To our knowledge, these results are the first to highlight the key value of T1 mapping in assessing pathological changes and aiding in the non-invasive diagnosis of renal involvement in AFD.

## 1. Introduction

Anderson–Fabry disease (AFD) is a rare X-linked inherited disorder resulting from a deficiency of α-galactosidase A (α-Gal A) activity, leading to the intracellular accumulation of glycosphingolipids such as globotriaosylceramide (Gb3) and its deacylated derivate globotriaosylsphingosine (lyso-Gb3). The reported prevalence of AFD ranges from 1 in 40,000 to 1 in 117,000; however, it seems to be underestimated. Progressive cellular accumulation of glycolipids results in cell dysfunction and several clinical manifestations, such as angiokeratoma, neuropathic pain, gastrointestinal manifestations, sweating abnormalities, and ear and eye lesions. The progression of the disease is characterized by multiorgan damage and can eventually lead to life-threatening kidney, heart and cerebrovascular system complications. Moreover, the activity level of α-Gal A, which can vary from diminished activity to total absence, is correlated with disease severity. AFD can be classified into the classic form (α-Gal A activity < 1% of mean normal), which affects hemizygous males and is characterized by multiorgan failure, severe clinical symptoms, early onset of the disease, and worse outcomes compared with late-onset affected males, characterized by higher levels of α-Gal A activity and less severe manifestations of the disease. Usually, affected females who typically exhibit α-Gal A activity levels, ranging from slightly reduced to nearly normal, have better prognoses despite the risk of developing a severe form of AFD due to X-chromosome random lyonization, in which some cells express the normal allele. Genetic modifiers, and epigenetic and environmental factors can affect the manifestation of the disease spectrum and contribute to variations within families [1]. 

Cardiac complications are common in patients with AFD, and cardiovascular magnetic resonance (CMR) and the T1 mapping technique are routinely used in the evaluation of patients with AFD as key tools for the assessment of cardiac involvement. Moreover, by the assessment of the alterations in myocardial parameters, such as the T1, T2 and T2* (star) relaxation times and extracellular volume (ECV), CMR allows the spatial visualization of quantitative changes in the myocardium. Intracellular and extracellular disturbances, such as glycosphingolipid accumulation in AFD, myocardial fibrosis or a combination of both (e.g., myocardial edema), can be identified by the changes in the CMR parameters. This technique represents an important breakthrough because it has been challenging to identify the myocardial involvement related to specific disease pathways, including AFD, without the use of invasive methods [2]. The use of a pulse sequence scheme, known as the modified Look-Locker inversion recovery (MOLLI) scheme, proposed by Messroghli et al., demonstrated to allow accurate in vivo T1 measurements and T1 mapping of the cardiac tissue within a single breath-hold and with high spatial resolution [3].

Although native T1 mapping has been extensively studied in myocardial fibrosis, few studies have evaluated its use in the context of renal fibrosis. Chronic kidney disease is considered one of the most important public health issues. Predicting the disease’s progression continues to pose a significant challenge, burdening patients and their families and placing an economic strain on society. Therefore, early identification of patients with rapidly declining renal function plays a crucial role in improving kidney health or slowing the progression of chronic kidney disease. Furthermore, researchers have tried to meet the need to find alternative methods for diagnosis, given that renal biopsy is invasive, often poorly tolerated by the patient, and has limitations due to the nature of the samples collected, which can result in non-accurate evaluations of overall kidney fibrosis. Over the past few decades, MRI has undergone rapid advancement, and it has been shown that native T1 mapping may enable the detection of kidney fibrosis, proving to be an effective non-invasive method for the earlier identification of patients most at risk for end-stage kidney disease [4]. Moreover, MRI can clearly show the anatomical differences between the renal cortex and medulla due to the shorter T1 relaxation times of the cortex. Corticomedullary differentiation is lost in several renal diseases as a consequence of interstitial damage, the accumulation of inflammatory infiltrates, and fibrosis of the renal parenchyma, ultimately leading to reduced glomerular filtration and end-stage kidney disease. Identification of T1 values of the renal cortex and medulla can be useful in determining the cortex/medulla ratio and, therefore, differentiating specific renal disease states, including renal fibrosis [5]. Wu et al. evaluated the utility of non-contrast-enhanced native T1 mapping of the renal cortex in assessing renal fibrosis in patients with chronic glomerulonephritis, demonstrating that native T1 mapping is a good diagnostic tool to evaluate kidney function and detect kidney fibrosis in this cohort [6].

Furthermore, Wei et al. conducted a prospective analysis of chronic kidney disease patients who underwent MRI examination, showing that the mean corticomedullary T1 difference and corticomedullary ratio values are capable of identifying patients with less than 25% and more than 50% of fibrosis, assisting clinicians in their decision-making process [7].

Based on these data, the use of MRI in patients with AFD was evaluated to identify the changes in renal T1 signal intensity. 

## 2. Materials and Methods

We conducted an observational retrospective analysis of 19 patients (Group A) with genetically confirmed AFD and normal renal function who underwent routine CMR examinations to evaluate cardiac involvement, using specific sequences that also included imaging of the left kidney. This group was compared with a similar control population (Group B) comprising 19 patients without AFD who had no signs of kidney disease and normal kidney function.

For the evaluation of cardiac and renal involvement in the study of Anderson–Fabry disease, we used the Philips D-Stream 1.5 T RM device, cardiac and respiratory gating, localization sequences in orthogonal sequences in orthogonal planes, functional sequences of the cine-balanced-SSFP orientation of 2, 3 and 4 chambers and the short axis, STIR-T2-black blood short axis sequences; T1-TSE sequences in 4 chambers; short axis dynamics during the administration of the IV contrast medium for the study of the “first pass” (Gadolinium), T1 Sh-MOLLI-Native without mdc, T1 after paramagnetic mdc for the study of mapping, PSIR-TFE in short axis, 2 and 4 chambers for the study of “Late Gadolinium Enhancement” and Q-flow breath-hold (phase contrast) for the study of flows. Because Fabry disease determines a microvascular dysfunction, which can be analyzed in the native T1 sequences for the mapping, we used specific ROIs (regions of interest) positioned at the level of the renal cortex, which allowed the quantification of both colorimetric and numerical values. We positioned three different ROIs at the renal cortex per patient and calculated the average values. Magnetic resonance imaging sequences can differentiate the renal cortex and medulla due to the shorter relaxation times (measured in ms) of the cortex. T1 mapping ROIs were then drawn for both the cardiac organ and the kidneys, and the values were recorded. Cardiac ROIs were acquired on the interventricular septum in order to analyze the myocardium. The renal ROIs were acquired, in collaboration with nephrologist colleagues, in the most cortical portion of the kidney possible, where diagnostic biopsies are performed.

The patients were selected among those who had undergone examinations for previous episodes of angina or arrhythmia, using the PACS (picture archiving and communication system) provided by the radiology unit of Policlinico Riuniti di Foggia, Italy, between August 2019 and February 2023. The study protocol conformed to the ethical guidelines of the Declaration of Helsinki and was approved by the institutional review board (Decision no. 158/CE/2023). A Spearman’s rank-order correlation was run to assess the relationship between the T1 values of the hearts and kidneys in Group A and between the kidneys in Groups A and B. 

## 3. Results

The preliminary analysis showed that the relationship was monotonic, as assessed by a visual inspection of a scatterplot, and no statistical differences were found in the gender, age and glomerular filtration rates between groups, as shown in Table 1.

**Figure 1:**
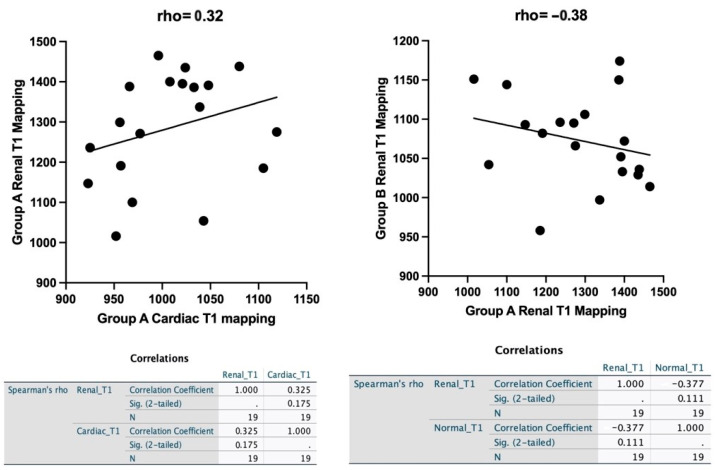
Spearman’s analysis shows a positive correlation between heart and kidney T1 mapping values in Group A and a negative correlation between kidney T1 mapping values in Group A and Group B.

Figure 1 shows that there was a non-statistically significant (*p* = 0.1) positive correlation between the heart and kidney T1 values in Group A (mean cardiac T1 mapping values 1007, 42 ± 57 ms; mean renal T1 mapping values 1284 ± 137 ms, rho = 0.32). 

More interestingly, we observed a negative correlation between the kidney values in both groups (Group A mean 1284 ± 137 ms; Group B mean 1073 ± 57 ms, rho = −0.38). The relationships were not statistically significant. Therefore, we cannot reject the null hypothesis and cannot accept the alternative hypothesis. 

Additionally, three patients exhibited a pseudonormalization of T1 values (mean value 1012 ± 18 ms) and a typical late gadolinium enhancement (LGE) of the basal segment of the infero-lateral wall of the left ventricle. Furthermore, the mean T1 values at the level of the postero-basal wall of the left ventricle showed lower mean T1 mapping values compared to the healthy patients (mean values 1007 ± 57 vs. 1059 ± 136 ms).

## 4. Discussion

To our knowledge, these results are the first to highlight the key value of MRI, particularly T1 mapping, in evaluating the pathological changes and facilitating the non-invasive diagnosis of renal involvement in AFD. Our results are of particular significance because they were obtained from a high-risk patient population with a rare disease. Due to efforts to screen patient groups with symptoms indicative of Fabry disease, three major “high-risk” groups have emerged: patients with cardiac involvement, stroke patients and kidney disease patients, considering that these patients are frequently diagnosed after irreversible organ damage. Kidney biopsy is the gold standard for diagnosis, and the patients could greatly benefit from early non-invasive diagnostic methods [8].

Cardiac involvement in AFD often appears early in life and typically manifests as left ventricular hypertrophy (LVH), resembling hypertrophic cardiomyopathy, arrhythmia, ischemia and abnormalities in the heart valves. The T1 value, defined as the longitudinal relaxation time, allows the identification of LVH, myocardial fibrosis and lipid accumulation without the requirement of a contrast agent.

**Figure 2:**
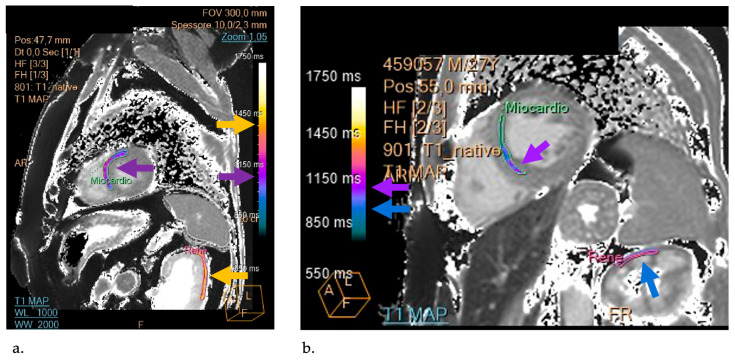
T1 cardiac and kidney values of representative patients. (**a**) Regions of interest (ROIs) at the level of the heart and renal cortex of AFD patients (mean values: 1048 vs. 1391 ms). Purple arrow: heart. Yellow arrow: kidney. (**b**) Regions of interest (ROIs) at the level of the heart and renal cortex of the control patient (mean values: 1002 vs. 958 ms). Purple arrow: heart. Blue arrow: kidney.

Additionally, a decreased native T1 value is considered a specific indicator for detecting myocardial glycosphingolipid accumulation before the onset of LVH, allowing for early recognition of cardiac dysfunction. In this study, we noted that there is a positive correlation between the T1 cardiac and kidney values in AFD patients, indicating that any cardiac longitudinal relaxation time corresponds to a higher renal T1 mapping value, as shown in the example in Figure 2. Exploring this correlation in depth will be the focus of our upcoming insights.

The most relevant issue of our work was the results obtained from the correlation between the renal T1 mapping values of the two groups. The renal longitudinal relaxation time (in contrast to what was observed in the myocardium) revealed an opposite fashion, showing higher T1 values in AFD patients compared to patients without kidney impairment. These findings are consistent with previous studies that have shown elevated renal parenchymal T1 mapping values in chronic kidney disease patients that may be attributed to pathological changes in the kidneys, such as interstitial inflammatory cell infiltration, edema or scarring. Moreover, fibrosis can lead to the loss of peritubular capillaries and subsequent hypoxic kidney injury, which may contribute to the rise in T1 values [9]. Furthermore, Peperhove et al. demonstrated that renal T1 values progressively increased with the severity of renal damage in kidney disease patients. Lastly, the renal medullary T1 value in healthy volunteers was significantly lower than in chronic kidney disease patients at all stages, suggesting that T1 mapping could be a valuable asset for the early detection of renal impairment [10]. Our findings suggest that the increased T1 values in AFD patients could be attributed to the presence of inflammatory infiltrates and edema in the tubular and glomerular compartments as a consequence of glycosphingolipid accumulation.

Moreover, our study showed that in three patients, a reduction in the T1 values was not consistently observed; instead, they exhibited pseudonormalization of the native T1 values, suggesting that they might have been in a progressive stage of the disease, characterized by scar formation. It has been proposed that myocardial involvement in AFD progresses through four different phases: beginning with a normal state, followed by a phase of reduced T1 values, then myocardial dysfunction and eventually heart failure with fibrosis. As the disease progresses, pseudonormalization of the native T1 values occurs as a sign of fibrosis [11]. Additionally, these patients also exhibited late gadolinium enhancement, which may be caused by a significant increase in the interstitial space or reduced gadolinium clearance from tissue, as seen in myocardial fibrosis [12].

The correlation between cardiac and kidney T1 mapping values could shed light on the potential renal involvement by AFD before the development of impaired kidney function and/or fibrotic lesions. In this context, similar to what occurs for cardiac involvement, MRI may be validated as the method of choice for suspected renal involvement in AFD once other potential causes of kidney disease have been excluded and before performing a renal biopsy. Indeed, it should be noted that T1 is also increased for fibrosis/scar because the collagen matrix of scars augments the interstitial space and water content and for amyloidotic deposits [13]. Interestingly, none of our patients show these features.

Potential study limitations include its retrospective nature of data collection, which exposed the study to confounding by measurement bias and unmeasured factors, the small number of patients, and the single-center study. However, AFD is a rare disorder, and a limited number of patients are expected. Thus, a prospective study with larger cohorts could mitigate these limitations. 

## 5. Conclusions

In summary, the diagnosis of AFD is often missed or delayed, and the identification of patients who may have the disease is crucial for the patient and their family members since they may be candidates for enzyme replacement therapy before organ failure. The high sensitivity of MRI in analyzing early kidney alterations in AFD could allow for an accurate assessment of organ impairment without needing invasive investigations, like kidney biopsy. Our results suggest a correlation between cardiac and renal involvement in AFD patients compared to the general population, leading us to suspect kidney involvement due to AFD despite normal kidney function. This represents the major strength of our study and should be configured as a feasibility study for future research.

## Figures and Tables

**Table 1:** Characteristics of the population.

	Group A (AFD Patients)	Group B (Control Population)	*p*-Value
Number (*n*)	19	19	
Gender (% male)	52.6%	57.8%	ns *
Age (years)	57.38 ± 17.26	53.38 ± 19.26	ns
GFR (mL/min)	96.21 ± 9.24	94.15 ± 10.11	ns
Comorbidities			
Diabetes Mellitus (*n*)	0	4	
Hypertension (*n*)	6	10	
Left Ventricular Hypertrophy (*n*)	12	8	
Previous Angina (*n*)	0	10	
Arrhythmia (*n*)	2	9	

* statistically non-significant.

## Data Availability

The datasets generated during and analyzed during the current study are available from the corresponding author upon reasonable request.
